# Exploring SPARC over Other Exerkines/Myokines: A Strategic Approach Towards Novel Exercise-Mimicking Therapies

**DOI:** 10.3390/biomedicines14020302

**Published:** 2026-01-29

**Authors:** Abdelaziz Ghanemi, Mayumi Yoshioka, Roseane de Fátima Guimarães, Jonny St-Amand

**Affiliations:** 1 Department of Physical Activity Sciences, University of Quebec in Trois-Rivières, Trois-Rivières, QC G8Z 4M3, Canada; abdelaziz.ghanemi.1@ulaval.ca (A.G.); roseane.guimaraes@uqtr.ca (R.d.F.G.); 2 Functional Genomics Laboratory, Endocrinology and Nephrology Axis, CHU de Québec-Université Laval Research Center, Québec, QC G1V 4G2, Canada; mayumi.yoshioka@crchudequebec.ulaval.ca; 3 Faculty of Pharmacy, Laval University, Québec, QC G1V 0A6, Canada; 4 Centre de Recherche en Organogénèse Expérimentale/LOEX, Regenerative Medicine Division, CHU de Québec-Université Laval Research Center, Québec, QC G1J 1Z4, Canada; 5 Department of Mechanical Engineering and Division of Medical Sciences, University of Victoria, Victoria, BC V8P 5C2, Canada; 6 Department of Molecular Medicine, Faculty of Medicine, Laval University, Québec, QC G1V 0A6, Canada

**Keywords:** secreted protein acidic and rich in cysteine (SPARC), myokines, exerkines, exercise, therapies

## Abstract

Identifying novel therapeutic targets for diseases and health conditions represents an important step towards new therapies. Functional genomics and proteomics represent promising tools in identifying such pharmacological targets. Within this context, numerous biomolecules have been characterized as exercise-induced (exerkines) in muscles (myokines). In this article, we present illustrative examples of why secreted protein acidic and rich in cysteine (SPARC) would be a “leading candidate” myokine/exerkine for exploring exercise-induced genes/proteins to develop therapies, clarify mechanisms, and improve various aspects of biomedical research, including population health.

Exploring pharmacological options that are based on mimicking the endogenous effects of the biomolecules has led to therapeutic tools such as insulin [1,2], erythropoietin [3], and glucocorticoids [4]. Within this context, it is important to select among the biomolecules—with similar effects or produced under similar conditions—those that seem most suitable to pursue for potential pharmacological/therapeutic exploration for clinical applications.

Exercise-related molecules represent a perfect modern example of this concept. Exercise has been studied and described as a “panacea” [5,6,7]. Indeed, exercise benefits are various and include reducing adiposity/body fat [8,9], osteopenia improvement [10], improving metabolic functions, mitochondrial biogenesis, and glucose tolerance [11,12] and glycemia [8,13], muscle development, oxidative phosphorylation (OXPHOS)/mitochondrial functions [14], increasing high-density lipoprotein cholesterol [8], improving immunity [15,16] and cancer prevention [17,18].

Therefore, it is an interesting medical purpose to mimic the effects of exercise using endogenous molecules known as exerkines, which are released following exercise [19]. The focus has been more specifically on myokines, which are muscle-released exerkines [20,21]. The therapeutic/clinical exploration of these molecules would specifically benefit individuals who need exercise effects but are unable to perform them due to physical disability, aging, or hospitalization, as well as patients who need the same effects as an adjuvant to improve their health or optimize therapies they already have. Thus, providing a therapeutic option for these individuals to overcome such challenging situations.

Herein, we briefly explain the rationale for focusing on secreted protein acidic and rich in cysteine (SPARC) among other myokines. SPARC, also known as osteonectin and BM-40 [22], is a 32 kDa matricellular secreted non-collagenous glycoprotein [23,24,25,26]. It interacts with extracellular matrix (ECM) proteins and has pleiotropic functions [23,25]. It also exhibits a calcium-binding [27] and collagen-binding [28,29] properties that impact its chemical and physical bioreactivity. Initially, SPARC was identified through both functional genomics and proteomics studies. We started focusing on SPARC following a functional genomics-based study (differentially expressed transcripts) that identified SPARC expression as the most exercise-induced gene [8].

The fact that SPARC shows a significant increase in expression suggests important implications for the exercise-induced phenotype, leading to the hypothesis that the exercise-induced effects/phenotype are (at least in part) SPARC-induced. The following hypothesis is that injecting SPARC could lead to an exercise-like effect(s)/phenotype. This would be via mimicking a physiological state rather than introducing an exogenous effect with potential unknown (and even harmful) consequences/effects. Thus, reducing the chances of side effects. Studies have already reported the benefits of injecting SPARC or increasing its expression (genetic modifications or exercise) that include tumorigenesis suppression [30], muscle strength increases, and adiposity decreases [31].

In addition, SPARC is expressed in many tissues, which will make therapeutic SPARC interact with those tissues’ physiological-like effects rather than “side effects”. SPARC also interacts via various intracellular pathways, which provide various options via SPARC-based pharmacology for molecular pharmacology against various diseases [32]. Within this context, SPARC properties have been associated with bone density [33], cellular adhesion promotion [25], tissue regeneration [34], angiogenesis [35], potential coronavirus disease-2019 (COVID-19) management [36], tissue remodeling [37], adipocytes remodeling, muscle metabolism [38], cancer management [39], and fibrillogenesis regulation [29]. Therefore, SPARC has been suggested as an anti-aging therapy (candidate) and even as an “exercise substitute” [40] including for immobilization-related muscle atrophy in elderly patients [41]. Moreover, its properties and expression pattern point to SPARC as a molecular biomarker for physiological and pathological processes such as exercise, obesity, and cancer. [42,43] as well as a molecular tool to optimize personalized medicine [44,45]. SPARC impacts not only muscles but also metabolism and adiposity, and it is produced not only by muscle but also by various tissues, suggesting additional effects and further therapeutic possibilities yet to be explored.

On the other hand, other excerkines or metabolic-related factors, some of which are already tested in clinical trials, have properties that allow us to still consider SPARC preferable to them. For instance, the literature reports that apelin, which can be exercise-induced [46], reverses age-associated sarcopenia [47]. Apelin and irisin are considered exercise-related effective actors in sarcopenic obesity [48], and clinical trials and preclinical phase of sarcopenia studies involving apelin and irisin are ongoing [49]. Unlike these two molecules, SPARC properties extend beyond sarcopenia and muscle effects, as it also benefits metabolism, regeneration, and immunity. For fibroblast growth factor-21 (FGF21), which has therapeutic potential for metabolic disorders [50], it may have side effects due to its multiple sites of action [51]. Even with therapeutic benefits and compared to SPARC, which is explored in various contexts, FGF21 and its therapeutic exploration remain mainly within metabolic contexts [50]. The same remark applies to brain-derived neurotrophic factor (BDNF), which is explored mainly in neurodegeneration [52,53,54]. For leptin, in spite of its beneficial metabolic effects, the issue of leptin resistance [55] limits its therapeutic potential.

These excerkine- and metabolic-related factors illustrate properties (both positively and negatively) of biomolecules that could—similarly to SPARC—be potential candidates but would have, unlike SPARC, either less therapeutic benefits, increased side effects, or reduced/limited efficiency. Moreover, compared with other myokines, SPARC has several advantages. Mainly, the diverse effects/properties it has (metabolic, cell growth, anti-cancer, anti-inflammation, muscle development, etc.). Such effects make SPARC a candidate for polypharmacology, targeting more than one condition with SPARC (instead of monotherapy or polytherapy for each condition) while preserving all the therapeutic benefits of such an approach.

Importantly, SPARC is—compared to the other myokines—what increases the most following exercise [8] suggesting, once again, its key roles in the exercise-induced benefits, which are the phenotype we aim to mimic in order to achieve therapeutic goals. The choice to focus on exploring SPARC is to provide therapeutic tools with various therapeutic effects across different tissues, for different diseases, and under various circumstances, mimicking endogenous effects (rather than introducing novel exogenous molecules) and minimizing the likelihood of side effects. However, depending on the situations and the specific targeted effects/tissues, we can choose other molecules or a combination of molecules for a more targeted/specific effect. SPARC would primarily confer a broader effect with multiple beneficial properties rather than a single effect on a single tissue.

Moving towards the possibility of SPARC administration as a therapy, it would most likely require injectable delivery, for which details (route of administration, dosing regimen, injection frequency, and overall therapeutic strategy for the use of SPARC) have yet to be studied. Regarding potential pharmacovigilance, the key aspects would primarily relate to the injection protocol (dosage, frequency, tissue distribution, etc.) rather than the protein itself. Indeed, as SPARC is an endogenous protein, its usage as a therapy would limit the risk of side effects. Side effects may result from differences in SPARC pharmacokinetics, dose/exposure, and tissue distribution between SPARC injection and exercise-induced endogenous secretion. Potential side effects could be related to SPARC’s chemical properties (calcium-binding and collagen-binding), which could lead to tissue calcification or fibrosis. The starting point of such dose-effect studies would be the physiological plasma SPARC concentration (e.g., 11.72 ± 4.47 μg/L [56]). Furthermore, SPARC-based therapy can be developed to encompass pharmacological or biomedical approaches targeting SPARC-related pathways or aiming to increase SPARC expression in specific tissues towards a more targeted treatment with fewer side effects.

In addition, SPARC properties, physiology-dependent and pathology-dependent properties position it as a diagnosis/stratification tool both in personalized medicine as well as for population health studies. Indeed, SPARC expression could reflect—for instance—the efficacy of an exercise in vivo and would allow us to predict how individuals would benefit from a long-term exercise-based therapy.
